# Interactions between special education teachers and children with chronic complex conditions: A qualitative study

**DOI:** 10.12688/f1000research.129122.1

**Published:** 2023-01-09

**Authors:** Haruo Fujino, Megumi Matsumoto, Aya Mieno

**Affiliations:** 1 Department of Child Development, United Graduate School of Child Development, Osaka University, Yamadaoka 2-2, Suita, Osaka, 5650871, Japan; 2 Department of Special Needs Education, Oita University, Dannoharu 700, Oita, 8701192, Japan; 3 Department of Behavioral Neurology and Neuropsychiatry, Osaka University United Graduate School of Child Development, Osaka University, Yamadaoka 2-2, Suita, Osaka, 5650871, Japan; 4 Hita Special Needs School, Nishiarita 2941-1, Hita, Oita, 8771352, Japan

**Keywords:** chronic illness, children, special education, interaction, child health, profound intellectual and multiple disabilities

## Abstract

Background: The number of children with complex medical conditions has increased in recent decades. In this context, a complex chronic condition is characterized by multiple morbidities that require intensive or continuous health care according to the level of severity. Given their various health conditions, it is challenging to provide special education to these children, but there is still insufficient evidence regarding the practical experiences of educators. The aim of this study was to investigate special education teacher’s perceptions, experiences, and challenges while developing interpersonal relationships and communicating with children who have complex chronic conditions.

Methods: We recruited and interviewed 21 special education school teachers. The transcripts of the interviews were analyzed using thematic analysis.

Results: Our analysis revealed four themes, including “searching for the meaning,” “complex chronic conditions as a difficult reality,” “widening experience for the future,” and “priority for interacting with children.” These themes reflect the perceptions, experiences, and challenges of the special education teachers.

Conclusions: In cases where children have severe functional limitations, it is more challenging to understand child-teacher interactions. This highlights the importance of searching for meaning in educational practices used among children with complex chronic conditions. Our findings may provide helpful insight into the experiences and challenges faced by special educators who engage with these children.

## Background

The number of children with complex medical conditions has increased in recent decades, although the rate of prevalence varies based on the definition and study context, ranging from 5–30% ( Beers *et al.*, 2003). While various categorizations and definitions exist in the literature ( van der Lee *et al.*, 2007), complex chronic conditions (CCC) is defined according to the International Classification of Diseases ( Feudtner *et al.*, 2000; Feudtner *et al.*, 2014). In an earlier study, Feudtner *et al.* (2000) proposed the following definition for CCC:

“Any medical condition that can be reasonably expected to last at least 12 months (unless death intervenes) and to involve either several different organ systems or 1 organ system severely enough to require specialty pediatric care and probably some period of hospitalization in a tertiary care center.”

To date, the revised description of CCC covers various complex medical conditions, including neurological and neuromuscular disorders, cardiovascular disorders, congenital and genetic defects, and technology dependence (e.g., tracheostomy) ( Feudtner *et al.*, 2014). In this framework, an epidemiological survey in the United States found that approximately 10% of children had one or more CCC, while 2% had multiple CCC ( Bjur *et al.*, 2019). In Japan, relevant data show that around 20,000 students (~0.002% of children) with health disorders receive special education in accordance with their special health care needs ( MEXT, 2018). Although many children with severe CCC who receive inpatient treatment appear to attend special needs schools, educational statistics on this issue are currently unavailable in Japan. While the exact rate of prevalence depends on the population, it is clear that there is an increasing number of children with CCC, which has therefore become an important area of focus in medicine and education ( Feudtner *et al.*, 2014; O'Connor *et al.*, 2019).

Children with multiple CCC generally exhibit more severe functional impairment and limited participation because of their disorders. Moreover, some children with CCC and severe intellectual disabilities are classified as having profound intellectual and multiple disabilities (PIMD), which is characterized by very severe cognitive and neuromotor impacts ( van Timmeren *et al.*, 2016). For example, the causes of PIMD include genetic disease, brain malformation, oxygen deficiency, and/or intractable epileptic seizure. Such conditions vary across a range of factors, including severity, trajectory, multiple organ involvement, medical and technological assistance, and special needs to address psychological and developmental difficulties ( McPherson *et al.*, 1998; van der Lee *et al.*, 2007). Thus, children with CCC may have a variety of special needs that require more attention and educational support in the school setting.

### Educational challenges confronted by special education teachers who engage with children with CCC

Schools for sick children play cardinal roles in the education and development of those with health disorders that require long-term treatment or frequent hospitalization. Although inclusive education is emphasized in the general school setting, challenges still arise when educating children with CCC ( Seki *et al.*, 2017). As emphasized in special needs education, higher expertise and continuous learning are also expected in the Japanese educational system ( Isogai, 2017). Given these factors, those with CCC or severe conditions often receive individualized education at special needs schools that are located in or adjacent to hospitals.

Since communication and interpersonal relationships are essential components in child development, the Japanese special education system aims to establish both ( MEXT, 2017). However, teachers who educate children with CCC may have difficulty achieving these goals due to functional limitations that arise under complex medical conditions. Moreover, children with very limited functioning require special curricula on a daily basis as well as continued intervention to improve their arousal, positioning, motor function, and communication ( Yeh *et al.*, 2019). Despite the fact that scholars have identified a gap between expected educational standards and actual practice, there is still a lack of evidence on the difficulties and challenges experienced by teachers ( Taniguchi, 2011b). In addition to the perspectives of parents and children, it is essential to investigate those held by teachers, as these latter points of view can help clarify practical roles when educating students with chronic conditions ( Runions *et al.*, 2020). While previous studies have focused on general difficulties in this context, there is still a lack of evidence on specific challenges that arise when attempting to establish interpersonal relationships and communicate with children with CCC, especially in the Japanese educational system ( Takeda, 2006; Taniguchi, 2011a). To improve special education practices when teaching children with CCC, it is therefore critical to investigate how teachers themselves understand and experience interactions and communication difficulties.

### Aim

This study investigated current perceptions, experiences, and educational challenges that special education teachers encounter when developing interpersonal relationships and communicating with children with CCC. We first developed the following qualitative research questions:

• What difficulties do teachers have when interacting and communicating with children with CCC?
• How do teachers perceive their experiences and educational practices when engaging with children with CCC?

## Methods

### Study design

This study adopted a cross-sectional qualitative design with semi-structured face-to-face interviews conducted on an individual basis from August 2018 to February 2019.

### Participants

The study participants were teachers who provided special needs education to sick children in Japan. We set the following inclusion criteria: 1) experience teaching sick children and/or those with CCC and 2) work in special needs schools for health disorders. In cooperation with their school principals, we selected participants with diverse backgrounds (i.e., elementary, middle, and high school levels and years in experience). The potential participant teachers were selected by the school principals based on the teachers’ experience and backgrounds. Ultimately, we recruited a total of 21 participants from three schools for hospitalized children with health disorders, including 15 from School 1, two from School 2, and four from School 3. All 21 teachers participated in this study after the researchers fully explained the study. We selected School 1 because it was the only school for hospitalized children with health disorders in Oita prefecture, Japan, where this study was conducted. However, Schools 2 and 3 were located in Oita and Hokkaido prefectures and they were selected based on differences in the conditions of children therein.

More specifically, School 1 was a special needs facility for children admitted to an adjoining hospital. The conditions of their disorders varied, including internal organ diseases (e.g., kidney diseases), neuromuscular disorders, and neurological disorders. As for functioning, their restrictions ranged from mild (e.g., physical activities and food intake) to severe (e.g., multiple severe disabilities). Of note, the severe conditions included a permanent vegetative state caused by hypoxic encephalopathy or traumatic brain injury. School 2 was a special needs facility for students who received outpatient treatment in Oita, while School 3 was a special needs facility in Hokkaido (another northern area in Japan) that received inpatient children with muscular dystrophies.

Table 1 lists demographic information for the study sample. As shown, 10 (48%) of the participants were women. Across the sample, teaching experience ranged from eight to 40 years (median 19, interquartile range [IQR] 13–29), while experience with special needs education for children with health disorders ranged from less than one year to 35 years (median 6, IQR 3–7). As for grade level, their work included students from elementary to secondary school. Table 2 summarizes the conditions of children from relevant experiences of the teachers.

**Table 1.** Participant demographics.

Demographic information	n
Sex	
Women	10
Men	11
Age (years)	
20–39	4
40–49	10
≥50	7
Years of school teaching experience	
1–10	2
11–20	9
≥21	10
Years of experience with children with CCC	
0–5	8
6–10	10
≥11	3
Department	
Elementary	9
Lower secondary	4
Upper secondary	8

CCC: complex chronic condition

**Table 2.** Chronic conditions that participants observed in their teaching experiences.

Disorder categories	Specific disorders
Disabilities (diseases not specified)	Profound intellectual and multiple disabilities
	Severe motor and intellectual disabilities
Neurological disorders	Cerebral palsy
	Hypoxic ischemic encephalopathy
	Intractable epilepsy
	Congenital hypomyelinating leukodystrophy
Neuromuscular diseases	Muscular dystrophy
	Congenital myopathy
	X-linked myotubular myopathy
	Spinal muscular atrophy
Other conditions	Chronic kidney disease
	Cancer
	Crohn’s disease
	Obesity
	Trisomy 18

### Procedures

This study adopted a cross-sectional qualitative design with semi-structured face-to-face interviews, as conducted on an individual basis from August 2018 to February 2019. As such, we asked the participants about their practical experiences and difficulties when engaging with students with CCC. We transcribed and audio-recorded all interviews for analysis. To draw narratives from their experiences, we developed an interview guide to capture potential challenges and personal beliefs regarding communication and interpersonal practices with children with special health care needs. This included questions about difficulties that may arise during teacher-student interactions, restrictions, and personal thoughts and practices (e.g., “Please describe your experiences when teaching children with complex chronic conditions, including any challenges you confronted during communication and when developing interpersonal relationships”). Specifically, we developed the interview guide using knowledge about the domain of interest, a literature review, and educator opinions. Domain knowledge included the conceptualization of education for sick children, knowledge of the diseases of children with CCC, and resources in Japanese educational systems. Although we used this guide to elicit experiential narratives, we also encouraged the participants to talk freely about any relevant views, ideas, experiences, and challenges. The interviews lasted from 43 to 71 minutes (median 52). They were conducted by the authors and a research assistant in Japanese. A copy of the interview guide can be found under *Extended data* ( Fujino, 2022).

### Data analysis

We analyzed the interview data through a thematic analysis. This allowed us to identify themes pertaining to the beliefs and challenges expressed by participants when teaching children with CCC at special needs schools. Using the thematic analysis approach, researchers aim to explore experiences and perceptions based on qualitative data ( Braun & Clarke, 2006; Clarke & Braun, 2013; Terry & Hayfield, 2021). In this study, we familiarized ourselves with the interview data by repeatedly reading the transcripts. Prior to identifying themes, we inductively coded the transcripts to identify relevant features based on our study topic. We did not generate *a priori* codes. The first author coded the transcribed text, then discussed the coding with another team member to develop a codebook. Because the codes were modified during this discussion, the original text was recoded to confirm the data context. We ultimately finalized the themes and codes through this iterative process. As an exploratory analysis, we also investigated differences based on years of experience educating sick children. In these processes, we used MAXQDA 2022 (VERBI Software GmbH, Berlin, German), a software for qualitative data analysis, in coding and integrating the transcripts. After completing the qualitative data analysis in Japanese (the original language of the data), the excerpts were translated into English for presentation in this article.

The key assumption was that teachers may confront challenges when teaching children with CCC. In this context, challenges related to communication and interpersonal relationships may be central issues; moreover, these factors may be compounded by restrictions in the educational environment ( Takeda, 2006; Taniguchi, 2011a). The first author is a psychologist who works with children and adolescents with disabilities, focusing on psychological support and special educational needs. The second author is a clinical psychologist with experience providing psychological support to individuals with neurodevelopmental disabilities and mental disorders. The third author is a special education teacher.

### Ethical considerations

This study was carried out in accordance with the ethical standards set forth in the 1964 Declaration of Helsinki and its later amendments. The protocol was approved by Oita University Faculty of Education Research Ethics Committee (approval number: H30-001-013). We obtained written informed consent from all participants prior to study enrollment. Participants agreed to the following treatment of data: the materials obtained in the interviews will be partially reported but personally identifiable information will be processed, and the materials will not be publicly available. We removed any identifying information from the interview transcripts before presenting them in this manuscript.

## Results

We identified four themes from the narrative interview data, including “searching for the meaning,” “CCC as a difficult reality,” “widening experience for the future,” and “priority for interacting with children.” In the following subsections, we explain these themes and provide direct quotes from participants. As we did not identify clear relationships between the thematic contents and number of years educating children with CCC, we omitted this subgroup analysis from our discussions. Participant demographic data can be found under *Underlying data* ( Fujino, 2022).

### Searching for the meaning

This theme describes beliefs about teacher-child interactions. The central concept pertains to the “true” meaning of responses and interactions with children with CCC, especially among those with severe conditions. In particular, it can be difficult to interpret responses from children with minimal communicative abilities (i.e., recognized responses are very subtle). Although objective evaluations are required in professional practice and may be useful for identifying consistency in reactions, subjective interpretations are also essential when attempting to find the meaning of the interactions.

In regard to communication difficulties, the central experience pertained to how teachers comprehended subtle responses from children with CCC. Some participants discussed questions about this issue when describing interaction problems. For example, children with profound disabilities often have minimal physical functioning and may only be capable of slight movements in the fingers or eyes. At the same time, comorbid intellectual and/or sensory disabilities complicate the process of understanding their actions and responses. In such cases, teachers may have difficulty interpreting the meanings of ambiguous or weak responses:

*The truth could be different from what I am thinking, but I do it [interacting] based on my own interpretation of the situation … it is difficult to say whether some sort of interaction with the intention of communication was actually established. (…) If someone were to ask me how I really think about the situation, I have to admit that I am not sure. I really don’t know.* (P10, 6–10 years of experience with sick children)

From this perspective, the “true” meaning of subtle physical responses is unknown. More difficult cases occur when interacting with children who exhibit “ *hardly any such movements*,” at times in association with a disorder of consciousness. One participant said the following: “ *Although there are some of them who are like this [who react with small movements], there are also those who cannot even express themselves in this way. It becomes really difficult in such cases*” (P13, 6-10 years of experience for sick children). Teachers who successfully interact with students with CCC may experience a *“really a wonderful feeling.”* While the participants intricately explained their beliefs and emotional experiences in the context of interacting with children with children with CCC ( Table 3, quote 1), their attempts were not always successful, especially in cases where children had minimal functioning. For example, one participant said the following about interacting with children who had disorders of consciousness: “ *feels like I am talking to myself and putting on a one-man show.*” This seems to describe an overly exhausting practical experience.

**Table 3.** Searching for the meaning.

Quote	Participant, Years of experience with sick children	Transcript
1	P13, 6–10	“When there are moments when we [children and teachers] can enjoy together, those times when I can really feel that we are bonded to one another, I feel wonderful … when I can get a reaction out of them, it was really a wonderful feeling.”
2	P02, 6–10	“However, I don’t think it is the case that I don’t know anything. Well, I guess, strictly speaking, we have not established anything; but, I have been trying to communicate. I have been trying to make approaches and interact.”
3	P01, 1–5	“It’s hard to say without this kind of numbers and such. I am concerned that it may be just my own personal impression; but, in order to prevent this, we have been keeping records as part of our school’s study and checking to see if it is the teachers’ assumption, too, so I have come to understand that if we do it objectively in this way, we can be sure of it.”
4	P08, 6–10	“I just want to make it a little bit clearer as to when he is sleeping and when he is awake. Since we don’t know right now, we decided to define he is ‘awake’ when his heart rate is above 70, and ‘asleep’ when it is below 60. Since he is here in the hospital, his heart rate is recorded like this every two seconds, so I got the data once and made a chart for the week, and about 25% of the time during the week it was over 70; but, even after going that far, I still wasn’t sure if it was really appropriate or not.”
5	P12, ≥11	“I wonder if it would be good to do something like create a story together with the child … that’s what I thought as I was talking to you. However, there is no way for me to verify if it’s actually true, but maybe something changes when you interact with a child, thinking that that’s the case.”

Despite various difficulties during teacher-child interactions, the participants insisted that they made attempts ( Table 3, quote 2). To avoid subjective interpretations made solely based on impressions, objective observations or physiological responses (e.g., heart rate and SpO _2_) may also be used to assess the status and reactions of children ( Table 3, quotes 3–4). By contrast, several participants described the need to make their communications into “a story.” Such narratives are also related to difficulties and questions pertaining to their engagement with children, especially among those with minimal functioning. One participant described the meaning of an interaction with a child with disorder of consciousness, wherein the lack of “readable” responses made it difficult to conceive the existence of an interpersonal interaction. As an alternative to objective assessments of responses/reactions, the participants sought more subjective individualized “stories” to derive meaning from their practices ( Table 3, quote 5). One said the following:

*Thus, objectively there is something there, and if there should be a reaction to it, and I can’t react back at all… so, in that respect, that’s why, when it comes to how to interact, whether something is there or not could just be more a problem from my side. It could be that something really is there, and I just can’t read it. That’s why [I think] a “story” is necessary here. It’s not that I am able to communicate with a person because I understand, but they are already there, existing, and they have a story.* (P08, 6–10 years of experience with sick children)

Without any such meaning, the practice becomes “quite uninteresting.” Therefore, creating or finding “a story” is an essential aspect of making interactions more meaningful. This constitutes an element of support for teachers who engage with children with minimal functioning and/or CCC.

### CCC as a difficult reality

This theme describes experiences with children’s illnesses and the conditions of their disorders. The participants valued thinking about the children’s experiences with their own disease as well as how they experienced the disease as an observer. For example, teachers may experience a child’s disease progression, perhaps even leading to death. Such descriptions reveal unique concepts that emerge when working with severely ill children.

The conditions of disorders certainly affect functioning and create limitations for children. Those with severe conditions may need to live in the hospital for years. Given these factors, the participants strongly recognized their participatory limitations. One described some common environmental limitations and discussed how children may experience them:

*They are children who spend all their time in a really small space [hospital room], in a similar [flow of time]. It’s summer vacation now, so I go to see them sometimes, but every day that they don’t come to school, they’re often asleep even when I go to see them, during the day. It’s hard to see anything but the world on their own bed. (…) They spend a lot of time alone.* (P02, 6–10 years of experience with sick children)

As some CCC are progressive in nature, cases in which conditions worsen can be “the most difficult,” both for the teachers and children. In fact, teachers may avoid talking about the progressive nature of such diseases altogether ( Table 4, quotes 6 and 7). For example, one participant described their difficulty with a child who had begun to lose ambulation due to Duchenne muscular dystrophy (a genetic progressive muscular disorder) ( Table 4, quote 8). From the teacher’s perspective, the emotional impacts of disease progression were recognized as “tough” due to various emotional and behavioral issues. When children have life-threatening illnesses, teachers may even experience their death. In fact, several participants had experienced the death of children with CCC. Even when participants had relevant expertise, these cases were very difficult, often evoking negative emotions, psychological distress, and grief:

*I knew that [the death] would happen; after all, there are severely ill children in the program, and several students have died since I came to the school. I don’t experience that kind of thing much at other schools. That’s what I don’t like about this school. I have to say goodbye [to children]. Well, it’s hard.* (P10, 6–10 years of experience with sick children)

In addition to the medical condition itself, educational systems sometimes become barriers for teachers. Teachers make individualized educational plans for children; however, the educational practice with the children does not proceed as originally planned. Realization of the plan is often not possible. Sometimes, there are “a lot of days off,” and “things don’t go well in terms of their health.” Because variability of the functioning and disabilities changes the ways of interaction and communication, teachers are required to have different ways of interacting with each child ( Table 4, quote 9).

**Table 4.** Chronic complex condition as difficult reality.

Quote	Participant, Years of experience with sick children	Transcript
6	P14, 6–10	“I think the most difficult thing to do is to see how much the children with muscular dystrophy know about their disease. Well, I’ve never been asked that question, but if I were asked, like, ‘What’s my disease?’ … Well, I wonder how I would answer that question.”
7	P17, 1–5	“I am a little concerned about how much we should talk about the disease; well, if it is a child with muscular dystrophy, to what extent we should talk about the disease in accordance with their developmental stage. Yeah. In fact, I’ve never told him about his illness, even though it’s been months since I’ve seen him face-to-face …”
8	P15, ≥11	“In the case of the child with progressive disease, there were tremendous symptoms of shock, even at the earlier stages. When a child with Duchenne muscular dystrophy cannot walk, the symptoms of shock are so great that the child inevitably becomes violent, and stress is released …”
9	P07, 1–5	“Children with multiple disabilities have different ways of interacting with each other, different goals, and different purposes, or rather, different ways of communicating, so it is difficult to do everything at the same time. Even when we do activities together, we all have different ways of interacting with each other.”
10	P21, 6–10	“Medical staff’s ideas about care and welfare were different from those of them in education, and I think that they valued different aspects based on [the] medical care system. It took me a long time to understand the medical side of things.”

Cooperation with medical care was considered another major environmental barrier. Although different professionals or organizations must collaborate to support children with CCC, cooperative engagement between medical professionals was perceived as challenging: *“Really, I think it is a bit of a difficult issue as to what real cooperation is. I can’t say they are cooperating, but I can’t say they aren’t”* (P11, 6–10 years of experience with sick children). Meanwhile, differences in language, attitudes, and values can hinder interprofessional cooperation ( Table 4, quote 10).

### Widening experience for the future

This theme describes beliefs about children’s experiences in their daily lives, special education schools, and hospitals. Because of various environmental and hospital-related restrictions, the participants valued the idea of widening their experiences with children with CCC. For such children, essential developmental components included time and interaction with other children, enhanced motivation to interact with surrounding individuals, and improved communication.

A central concept of this theme is rooted in the fact that most children with severe CCC spend “most of their time in the hospital” to receive inpatient treatment. As a result, the range of their experiences and activities tends to be limited. Although the participants eagerly tried to create opportunities for interactions with other people, such efforts were often difficult due to environmental limitations, including the hospitalization itself:

*They don’t have a lot of opportunities to interact with people, and since they are basically one-on-one with the teacher, it’s hard for them to broaden their relationships with other people. I was thinking that it would be nice if I could do something to help them expand their relationships with others, such as students, [and] nurses at the hospital.* (P20, 1–5 years of experience with sick children)

Opportunities to interact with peers were considered essential, and therefore encouraged ( Table 5, quote 11). The participants hoped to widen interactions with other children and facilitate interactions with surrounding adults, as each student was used to being accompanied by a teacher ( Table 5, quote 12). However, it is also important to note that adult-student relationships sometimes hindered children from behaving independently or spontaneously ( Table 5, quote 13). For that reason, the participants carefully made their attempts: “ *[I tried] to help as much as possible, but sometimes, I also tried to wait until the patient was willing to move his/her hand as much as possible*” (P09, 6–10 years of experience with sick children).

**Table 5.** Widening experience for the future.

Quote	Participant, Years of experience with sick children	Transcript
11	P01, 1–5	“That child liked to interact with adults, as the adults were more likely to understand and talk to them. Nevertheless, we tried to make them talk to their peers of [the] same age as much as possible. If there were other sick children, I would have told them, for example, to help children who seemed to be having difficulty holding things … yes, I would have tried to encourage that child to build relationships.”
12	P13, 6–10	“Since the classes are limited, it is difficult for them to interact with other children, and we have to visit them. So, in the second year, I arranged for him to visit the school and participate in activities with other students in the same grade … so, I wanted the students to work on various things by themselves, since they were paired with adults.”
13	P21, 6–10	“I think that they have been protected by the relationship with their illness, and people around them took care of them … they spent a lot of time being cared for … and then, one day, they suddenly realized that they may lose initiative.”

From the future-oriented perspective, teachers value continued developmental goals for children, but those with CCC often take longer to achieve them, in some cases years. One participant said: “ *Sometimes it is the year to create a starting point, and other times it is the year when we have to produce an achievement*” (P02, 6–10 years of experience with sick children). Even if the teacher cannot identify significant improvements in targeted behaviors or performances, some progress may still result in future developmental gains for children with severe CCC. In the long-term context, such perceptions may support teachers who engage with these children.

### Priority for interacting with children

This theme describes beliefs about interactions and relationships with children with CCC. We also identified two subthemes. The first is “ *relationship as a basis for interacting with children*,” referring to the belief that a positive child-teacher relationship creates a fundamental basis for meaningful interactions and educational practices. The second is “ *child’s life after leaving the school/hospital*,” referring to the idea that the supportive relationship will end once the child graduates from school or is discharged from the hospital.

*Relationship as a basis for interacting with children*

Since relationships are viewed as a critical basis for interaction and communication, the participants valued the process of building them with children with CCC “ *rather than teaching any classes first.*” Given that illness directly affects the lives of these children, one participant pointed out the importance of giving them time to adapt to life in the hospital ( Table 6, quote 14), especially because this environment often increases anxiety.

Many hospitalized children have a range of conditions that require inpatient treatment. Thus, facing medical difficulties is also a critical aspect of building trusting relationships with those who have severe conditions. For children with life-threatening diseases such as cancer, teachers sometimes face suffering, but also share hopes, feelings, and emotions. While this was discussed as a particularly “ *hard and painful*” experience ( Table 6, quote 15), the participants continued to believe in the meaning of teachers maintaining their presence with children and working toward supportive relationships.

**Table 6.** Priority for interacting with children.

Quote	Participant, Years of experience with sick children	Transcript
14	P13, 6–10	“I think that … when a person becomes ill, it means that they can no longer do the things they used to normally do in their daily lives, so the mental part of it is something that we have to take into consideration. I think. Little by little, as we spend time in a lightly relaxed manner, gradually, you know, that they get used to us.”
15	P15, ≥11	“When the child was awake [and conscious], I would let the child do whatever he or she wanted to do until the end. In that sense, [teachers] are there to empathize with that state of the child and encourage and support what they want to do. In most cases, even just to let them know that the teacher is there, next to the bed, you may spend more time holding hands; to let them know that they are not alone. I was conscious to teach in a way that allowed me to convey such messages. So, it was common to see teachers crying for a while after returning to the staff room from the sickroom. After all, these are things that are hard and painful.”
16	P04, 1-5	“[Being sick], it’s hard things … hmmm. Would be nice if the illness could be cured. The most important thing is that, you know, I want them to get well and go home. I want them to do well at the local school where they were enrolled.”

*Child’s life after leaving the school/hospital*

The children were also seen as having life in future contexts. As most were enrolled at schools for hospitalized children, their attendance was not considered firm. Some remained for very short periods (e.g., two to several weeks), while others stayed much longer (e.g., six months to multiple years). As teachers are therefore given limited time to involve themselves with these children, they tend to think about their lives after school or the hospital. One participant said: “ *I guess the present situation is that temporary, a place to come*” (P08, 6–10 years of experience with sick children). In this sense, the children were viewed as going away for a short period of time, in contrast to schools where they would be expected to remain until graduation. According to another participant, continued relationships are associated with medical conditions that do not sufficiently improve for hospital discharge. Thus, leaving the school and ending the relationship implies that the child’s condition has improved ( Table 6, quote 16).

The concept of life after leaving school also contextualized a range of experiences outside the school setting. Both at the school for sick children and in the hospital, conditions were well-understood by teachers and other professionals, but efforts were often required to avoid their recurrence after leaving. One participant said: “ *Even though I am getting older and have been a teacher for a long time, I still have concerns about where doing this will lead [in the child’s future]*” (P11, 6–10 years of experience with sick children). In this regard, even experienced teachers may have difficulty predicting children’s long-term needs and incorporating them into their educational practices.

## Discussion

Focusing on areas of interaction and communication, this study investigated current perceptions, experiences, and educational challenges encountered by special education teachers who work with children with CCC. We identified four main themes, including “searching for the meaning,” “CCC as a difficult reality,” “widening experience for the future,” and “priority for interacting with children,” each of which summarizes aspects that are relevant to schools for sick children in the Japanese context.

### Meaning of interactions

This study focused on interactions and communications between teachers and children with CCC. The process of supporting sick children can generally act as a source of motivation and satisfaction for teachers ( Lopez & Corcoran, 2014; Małkowska-Szkutnik *et al.*, 2021), but our results suggest that interactions with children with CCC often lead to difficulties, especially in cases involving severe or complex conditions. To ensure that communication is reciprocal, teacher must focus on careful communicative approaches and responses when working with children with severe disabilities ( Fylkesnes & Ytterhus, 2021). However, this process can be extremely difficult when children have severe limitations, as suggested in this study. In cases where children cannot or do not respond, teachers may find it very difficult to receive tangible feedback on their efforts. In their practices with these children, our participants questioned and searched for the *meaning* of both their involvement and the employed educational approach. In such cases, objective evaluations may help clarify the state and usefulness of those approaches ( Nozaki & Kawasumi, 2013). In the same instance, one may therefore find it useful to discuss the issue from a value perspective (i.e., finding meaning and value in educational practices).

A theoretical consideration rooted in phenomenological perspectives on subjectivity and objectivity may provide insight into interactions with children who have minimal functioning ( Evensen, 2021). One direction of interaction and communication was taken from the perspective that communication is a form of pure technique. This makes children’s expressions more accessible to unfamiliar individuals. In turn, the same perspective is important for sharing with other individuals who are involved with the child (e.g., medical professionals), as also described by our participants. Another direction is offered by the perspective that considers contextual impressions and relational/interactional stimuli ( Evensen, 2021), which entails a more subjective nature of perceptions and interpretations. In sum, professionals who work in special needs education should integrate objective and subjective perspectives during their interactions with children, especially when searching for meaning in their practices.

### The challenge of CCC

CCC is a critical aspect that affects the challenges and difficulties encountered by special education teachers and their students. As several participants in this study described, teachers may experience cases in which the child mentally and physically collapses, perhaps leading to worsened health or death. Because they tend to have emotional involvement with these children, such relational aspects may lead to other challenging experiences ( Benigno & Fante, 2020; Hart & Garza, 2013; Małkowska-Szkutnik *et al.*, 2021; Steinke *et al.*, 2016). Teachers who witness physical and psychological pain in children may also incur psychological impacts due to their relational involvement. While developments in medical treatment have reduced the occurrence of such cases, they are still difficult for teachers to experience, and often characterize the educational practices they employ for sick children ( Hart & Garza, 2013). In these situations, teachers are required to be resilient and sufficiently cope with their emotional responses ( Benigno & Fante, 2020; Hart & Garza, 2013). To better position themselves for this, they may refer to professional experiences, train to improve coping strategies, and seek support from colleagues ( Małkowska-Szkutnik *et al.*, 2021).

To widen the child’s experience, teachers may also need to improve their own skills. As discussed above, teachers must adopt particular values or philosophies in their educational practices ( Hillel Lavian, 2015). In the absence of these elements, the process of educating students with complex needs may evoke strong emotional responses in teachers, potentially leading to emotional fatigue and burnout ( Hillel Lavian, 2015). To avoid this, special education teachers who work at in-hospital schools should seek further development by availing of support from colleagues, sharing beliefs relevant to the support of children, and altering their own teaching values ( Soejima *et al.*, 2020). In turn, this may support emotional adaptation and enhance their professional capacity.

In Japan, efforts to accumulate professional experience and improve self-efficacy are urgently needed in special education for sick children, as teachers currently have limited experience in this field ( Nagae, 2016; Takeda & Kasahara, 2001). In addition to general training for health disorders, specific training is required for some conditions, including rare genetic diseases and those associated with severe functional limitations ( Garvey *et al.*, 2020).

### Challenges in educational practice

Although peer interactions are essential features in educational practice, children at hospital-based schools tend to have limited opportunities for this. Meanwhile, environmental restrictions create educational issues ( Knauer *et al.*, 2015). In this study, teachers believed it was important for students to have opportunities to be with or interact with other children. Indeed, research has shown that opportunities for peer interactions are important in child-child interactions, even if their disabilities are severe and multiple in nature, as this may increase spontaneous attention or approaches toward other children ( Nijs *et al.*, 2016). At the same time, these interactions are expected to widen the experiences of children and facilitate their relational and emotional development ( Buysse & Bailey, 2016; Justice *et al.*, 2014). Despite the various environmental limitations, teachers should therefore help hospitalized children engage in peer interactions and value activities that widen the potential for social relationships.

Coordination with other professionals is a long-standing issue in special needs education for sick children. While interdisciplinary communication and cross-organizational relationships, particularly with medical professionals, are emphasized in literature, this is often a challenging practice at schools in many countries ( Ballard & Dymond, 2018; Małkowska-Szkutnik *et al.*, 2021; McClanahan & Weismuller, 2015; Pufpaff *et al.*, 2015). According to reports, the lack of a collaborative process is partly due to insufficient time for collaboration and inadequate interprofessional communication ( Garvey *et al.*, 2020). Moreover, cooperative engagements between individuals in different professional roles often lead to conflict, and are influenced by divergent opinions, leadership, and counseling abilities ( Weiss *et al.*, 2018). These issues should be discussed and negotiated based on the expectations and needs of stakeholders. Earlier education programs for interprofessional collaboration may also facilitate the development of a foundation for accepting and balancing the weakness and strengths of each professional.

### Implications for practice

Developments in communications technology may support the objective evaluation of children with profound disabilities. While some technologies help us communicate and interplay with children, the actual degree of technology use is influenced by whether the teacher perceives its value in special needs education ( Anderson & Putman, 2020). Therefore, it is still important to assess their values on how and what is used, as well as how the interactions are performed. In addition, continuous education for various learning approaches may stimulate the skills and creativity of teachers in the context of individualized and differentiated teaching ( Weiss *et al.*, 2018). To develop expertise in education for seriously ill children, this emphasizes the need to continue accumulating experience in the field. In this regard, seasoned professionals may serve as role models to help teachers with limited experience ( Soejima *et al.*, 2020; Takeda & Kasahara, 2001).

### Limitations

This study also had some limitations. First, the range of children’s characteristics may strongly affect the difficulty experienced by special education teachers. Several of our participants experienced educational practices with children with profound disabilities (e.g., a disorder of consciousness), while others experienced relatively acute conditions. In this regard, the characteristics of disorders and/or functioning may have affected the findings. A more focused approach among a more targeted population may reveal additional context-dependent issues. Second, we did not include the parents of children with CCC. Because interactions are based on phenomena constructed by two or more individuals, the involvement of individuals in these positions may reveal a more complex nature. It may also be important to consider that discrepancies exist between children, parents, and teachers ( Maciver *et al.*, 2019; O'Connor *et al.*, 2016). Third, the transferability of this study may be limited in certain conditions. As the education systems for children with CCC and teacher training may differ between countries, the experiences of teachers may vary by location. However, the core themes identified in this study should be similar in other settings (e.g., difficulty interpreting limited responses from children and facing difficult conditions). Despite these limitations, this study provides valuable insight into the conditions experienced by teachers working in education for children with CCC.

## Conclusion

This study conducted interviews with special education teachers to investigate the qualitative aspects of experiences with children with severe CCC. In sum, our findings suggest that teaching beliefs and values are strongly connected with actual practice. At the same time, severe functional limitations in children often lead to major challenges when professionals attempt to find meaning in interactions between teachers and children with CCC. This study may clarify important challenges and experiences encountered by special education teachers who work with these children.

## Data Availability

The raw transcripts from the interviews are not publicly available to protect participants' privacy. Excerpts of the transcripts were included in the article. Transcripts in Japanese (the original language of the interview) are available from the corresponding author upon reasonable request at fjinoh@kokoro.med.osaka-u.ac.jp. Contact details, aims to use the transcripts, and data security management will be required. OSF: Interactions between teachers and children with CCC. https://www.doi.org/10.17605/OSF.IO/WQNFV. ( Fujino, 2022) This project contains the following underlying data:
- Participant_demographic.csv This project contains the following extended data:
- Interview_guide.pdf

## Reporting guidelines

OSF: Interactions between teachers and children with CCC. https://www.doi.org/10.17605/OSF.IO/WQNFV. ( Fujino, 2022)

- SRQR_checklist.pdf

Data are available under the terms of the Creative Commons Attribution 4.0 International license (CC-BY 4.0).
