# Supplementary material for: Interactions between special education teachers and children with chronic complex conditions: A qualitative study
Source: F1000Res. 2024 May 22;12:23. Originally published 2023 Jan 9. [Version 3] doi: 10.12688/f1000research.129122.3 (PMC11079580; doi:10.12688/f1000research.129122.3)
Supplement: Peer review history

# Reviewer report: Reviewer response for version 3

**DOI:** 10.5256/f1000research.166794.r281458

**Published:** 2024-09-18

**Authors:** Yuta Koto

**Affiliations:** 1 Faculty of Nursing, Graduate School of Nursing, Kansai Medical University, Osaka, Japan

Thank you for your modification. I confirmed that no further revisions were necessary.

Is the work clearly and accurately presented and does it cite the current literature?

Yes

If applicable, is the statistical analysis and its interpretation appropriate?

Not applicable

Are all the source data underlying the results available to ensure full reproducibility?

Partly

Is the study design appropriate and is the work technically sound?

Partly

Are the conclusions drawn adequately supported by the results?

Partly

Are sufficient details of methods and analysis provided to allow replication by others?

Partly

Reviewer Expertise:

Nursing; Disabilities

I confirm that I have read this submission and believe that I have an appropriate level of expertise to confirm that it is of an acceptable scientific standard.

# Reviewer report: Reviewer response for version 3

**DOI:** 10.5256/f1000research.166794.r281460

**Published:** 2024-05-23

**Authors:** Angelica Moè

**Affiliations:** 1 University of Padova, Padua, Italy

The AA improved the Introduction by adding more recent quotations. However the novelties of the research should be emphasized more. Hence I would suggest

 1) to stress more the novelty of your research

 2) to provide explicit predictions based on the literature gaps 

 Then, in the Discussion

 3) I would emphasize more the role played by teacher enthusiasm in favoring positive relationships and being supportive (e.g., Moè & Katz, 2022 [Ref 1])

Is the work clearly and accurately presented and does it cite the current literature?

Partly

If applicable, is the statistical analysis and its interpretation appropriate?

I cannot comment. A qualified statistician is required.

Are all the source data underlying the results available to ensure full reproducibility?

Partly

Is the study design appropriate and is the work technically sound?

Yes

Are the conclusions drawn adequately supported by the results?

Partly

Are sufficient details of methods and analysis provided to allow replication by others?

Yes

Reviewer Expertise:

Teacher motivation and well-being

I confirm that I have read this submission and believe that I have an appropriate level of expertise to confirm that it is of an acceptable scientific standard, however I have significant reservations, as outlined above.

## References

[1] : Need satisfied teachers adopt a motivating style: The mediation of teacher enthusiasm. *Learning and Individual Differences* .2022;99: 10.1016/j.lindif.2022.102203 10.1016/j.lindif.2022.102203

# Reviewer report: Reviewer response for version 2

**DOI:** 10.5256/f1000research.159878.r269182

**Published:** 2024-05-08

**Authors:** Yuta Koto

**Affiliations:** 1 Faculty of Nursing, Graduate School of Nursing, Kansai Medical University, Osaka, Japan

**General comments**

 This study identifies the experiences and challenges of teachers involved in the education of children with chronic illnesses and disabilities. The results are valuable in an area where there is a lack of accumulated knowledge. Another strength of this paper is that it draws information from multiple facilities and regions. The revisions have already been made based on comments from two reviewers, and a certain level of quality has been maintained. However, I believe that additional information on trustworthiness would be needed to ensure the rigor of the study.

**Recommendation for revision**

1. Background. MEXT2018 is cited as the current status of CCC children in Japan, but I don't think it is appropriate. At least, the data of 20,000 children cannot be referenced.
2. Method Participants. The first inclusion criterion is written as sick children and/or children with CCC. Does this mean that teachers with experience only with sick children in general, not CCC, would be included at planning phase?
3. Method Participants. The authors indicate that teachers with minimal experience were excluded, but what specific criteria were used to define minimal experience? Was it the length of experience or the number of children in their charge?
4. Procedure. The authors stated that they conducted semi-structured interviews and developed an interview guide. However, they also state that they were not used. In other words, it is unclear whether the authors conducted unstructured or semi-structured interviews.
5. Data analysis. The characteristics of the first, second, and third authors are described. This is an important matter for the trustworthiness of qualitative research. However, one research assistant also participated in the conduct of the interviews. Is this research assistant similar to the characteristics of the first and third authors? Differences in experience in data collection would affect rigor.
6. Results. In the analysis section, the authors note that they investigated differences based on the participant's years of experience. The results are written along four themes, but what were the results obtained from the different years of experience?

Is the work clearly and accurately presented and does it cite the current literature?

Yes

If applicable, is the statistical analysis and its interpretation appropriate?

Not applicable

Are all the source data underlying the results available to ensure full reproducibility?

Partly

Is the study design appropriate and is the work technically sound?

Partly

Are the conclusions drawn adequately supported by the results?

Partly

Are sufficient details of methods and analysis provided to allow replication by others?

Partly

Reviewer Expertise:

Nursing; Disabilities

I confirm that I have read this submission and believe that I have an appropriate level of expertise to confirm that it is of an acceptable scientific standard, however I have significant reservations, as outlined above.

# Response

**Published:** 2024-05-19

**Authors:** Haruo Fujino

**Affiliations:** Osaka University, Japan

**Response to the reviewers**

**General comments**

 This study identifies the experiences and challenges of teachers involved in the education of children with chronic illnesses and disabilities. The results are valuable in an area where there is a lack of accumulated knowledge. Another strength of this paper is that it draws information from multiple facilities and regions. The revisions have already been made based on comments from two reviewers, and a certain level of quality has been maintained. However, I believe that additional information on trustworthiness would be needed to ensure the rigor of the study.

**Response:**

 Thank you for helpful feedback on our manuscript. Based on the reviewers’ comments, we have revised the manuscript. Please refer to our responses for the specific comments.

**Recommendation for revision**

 1.           Background. MEXT2018 is cited as the current status of CCC children in Japan, but I don't think it is appropriate. At least, the data of 20,000 children cannot be referenced.

**Response:**

 In the introduction section, a sentence citing “MEXT: Special needs education in Japan, 2018” states as follows: In Japan, relevant data show that around 20,000 students (~0.002% of children) with health disorders receive special education in accordance with their special health care needs ( MEXT, 2018).

 This sentence provides information about the number of students with health disorders who received special education, not the number of students with CCC. As the reviewer described, the number of children with CCC is not currently available in Japan. Therefore, the following sentence in the section states as follows: Although many children with severe CCC who receive inpatient treatment appear to attend special needs schools, educational statistics on this issue are currently unavailable in Japan.

 2.           Method Participants. The first inclusion criterion is written as sick children and/or children with CCC. Does this mean that teachers with experience only with sick children in general, not CCC, would be included at planning phase?

**Response:**

 Thank you for pointing it out. Actually, participants were recruited if they had experience teaching “sick children”, because detailed experience about students with CCC was able to be confirmed only after a detailed interview for their teaching experiences. Therefore, we modified the sentence:

In Participants section,

 The participants were recruited based on their experience in teaching sick children (i.e., health disorders). We set the following inclusion criteria: 1) experience teaching children with CCC and 2) work in special needs schools for health disorders. The first criterion was confirmed after the interview because we obtained detailed information on their teaching experience in the interviews.

 All 21 teachers participated in this study and were confirmed that they had experience in teaching children with CCC.

 3.           Method Participants. The authors indicate that teachers with minimal experience were excluded, but what specific criteria were used to define minimal experience? Was it the length of experience or the number of children in their charge?

**Response:**

 Thank you for your clarification on the minimal experience of the teachers. The experience was defined based on the duration, i.e., four months (a school term in Japan).

 Based on the reviewer’s comment, we clarified the information.

In Participants section,

 Teachers with minimal experience in this area (i.e., less than four months) were not recruited.

 4.           Procedure. The authors stated that they conducted semi-structured interviews and developed an interview guide. However, they also state that they were not used. In other words, it is unclear whether the authors conducted unstructured or semi-structured interviews.

**Response:**

 Sorry for your confusion. In response to the reviewer’s comment, “it” indicated a pre-testing or pilot interview to evaluate the interview guide. Unfortunately, the sentence was accidentally deleted by copy-editing. We corrected the sentence as originally described.

In Procedures section,

 “A pilot-testing of the guide was not performed for this study.”

 5.           Data analysis. The characteristics of the first, second, and third authors are described. This is an important matter for the trustworthiness of qualitative research. However, one research assistant also participated in the conduct of the interviews. Is this research assistant similar to the characteristics of the first and third authors? Differences in experience in data collection would affect rigor.

**Response:**

 Another interviewer was a special education teacher similar to the third author. Based on the reviewer’s suggestion, we have added the following:

In Procedures section,

 The interviewers discussed the research aim, topic, educational systems, and possible challenges when teaching children with CCC several times. The first author provided training (about 4.5 hours) for the current study to maintain the quality of the interviews.

 6.           Results. In the analysis section, the authors note that they investigated differences based on the participant's years of experience. The results are written along four themes, but what were the results obtained from the different years of experience?

**Response:**

 We have described that “we did not identify clear relationships between the thematic contents and number of years educating children with CCC.” Although we cannot exclude the possible association between the themes and experience years in teaching, the number of years may not be the best index for capturing teachers’ abilities or depths of experiential knowledge.

 We have now revised the limitations section as follows: 

 As we did not find significant differences by the years of experience with children with CCC, the index may not be the best measure for capturing teacher’s abilities or depths of experiential knowledge.

# Reviewer report: Reviewer response for version 1

**DOI:** 10.5256/f1000research.141784.r209534

**Published:** 2023-11-27

**Authors:** Angelica Moè

**Affiliations:** 1 University of Padova, Padua, Italy

The MS presents a study conducted with special education teachers to catch their experiences with children with severe complex chronic conditions (CCC).

 The topic is interesting and it deserves investigation for its practical implications and the paucity of studies on it.

 Hence. I am positive about indexing pending the revisions listed below.

 Background. I would report more recent data on the prevalence of the diseases. They refer to 20 years ago: Beers et al., (2003). Overall, I would refer mostly on recent literature. Maybe specific reference to similar studies should be made more explicit by adding texts (as it is now it appears pretty short).

 Discussion. I would report in more detail which programs or interventions suggested for helping teachers in facing the burdens due to interacting with CCC children.

 Generalization: They found those results in a specific context. Do the AA think they can be applied worldwide?

 Overall. I would refer to a specific theory. Maybe the SDT theory regarding the autonomy-support vs. control could help (e.g., Ryan & Deci, 2020) because it states different supportive or less functional modalities (e.g., Moè et al., 2022). Referring to self-efficacy could be a further option (e.g., Kasalak & Dagyar, 2020).

 Suggested references:

 Kasalak, G., & Dagyar, M. (2020). The relationship between teacher self-efficacy and teacher job satisfaction: A meta-analysis of the teaching and learning international survey (TALIS). Educational Sciences: Theory and Practice, 20(3), 16-33.

 Moè, A., Consiglio, P., & Katz, I. (2022). Exploring the circumplex model of motivating and demotivating teaching styles: The role of teacher need satisfaction and need frustration. Teaching and Teacher Education, 118, 103823.

 Ryan, R. M., & Deci, E. L. (2020). Intrinsic and extrinsic motivation from a self determination theory perspective: Definitions, theory, practices, and future directions. Contemporary Educational Psychology, 101860.

 I wish the AA the best with their research!

Is the work clearly and accurately presented and does it cite the current literature?

Partly

If applicable, is the statistical analysis and its interpretation appropriate?

I cannot comment. A qualified statistician is required.

Are all the source data underlying the results available to ensure full reproducibility?

Partly

Is the study design appropriate and is the work technically sound?

Yes

Are the conclusions drawn adequately supported by the results?

Partly

Are sufficient details of methods and analysis provided to allow replication by others?

Yes

Reviewer Expertise:

Teacher motivation and well-being

I confirm that I have read this submission and believe that I have an appropriate level of expertise to confirm that it is of an acceptable scientific standard, however I have significant reservations, as outlined above.

## References

[1] : The Relationship between Teacher Self-Efficacy and Teacher Job Satisfaction: A Meta-Analysis of the Teaching and Learning International Survey (TALIS). *Theory and Practice* .2020;20(3) : Reference source

[2] : Exploring the circumplex model of motivating and demotivating teaching styles: The role of teacher need satisfaction and need frustration. *Teaching and Teacher Education* .2022;118: 10.1016/j.tate.2022.103823 10.1016/j.tate.2022.103823

[3] : Intrinsic and extrinsic motivation from a self-determination theory perspective: Definitions, theory, practices, and future directions. *Contemporary Educational Psychology* .2020;61: 10.1016/j.cedpsych.2020.101860 10.1016/j.cedpsych.2020.101860

# Response

**Published:** 2023-12-12

**Authors:** Haruo Fujino

**Affiliations:** Osaka University, Japan

**Reviewer Report**

*27 Nov 2023 | for Version 1*

*Angelica Moè, University of Padova, Padua, Italy*

*APPROVED WITH RESERVATIONS*

*General comments:*

*The MS presents a study conducted with special education teachers to catch their experiences with children with severe complex chronic conditions (CCC).*

*The topic is interesting and it deserves investigation for its practical implications and the paucity of studies on it.*

*Hence. I am positive about indexing pending the revisions listed below.*

**Response:**

 We appreciate your encouraging comments on our manuscript.

*1. Background. I would report more recent data on the prevalence of the diseases. They refer to 20 years ago: Beers et al., (2003). Overall, I would refer mostly on recent literature. Maybe specific reference to similar studies should be made more explicit by adding texts (as it is now it appears pretty short).*

**Response:**

 Based on the reviewer’s suggestion, we added a recent report from the United States (Ghandour et al., 2022).

 In Background section,

 The number of children with complex medical conditions has increased in recent decades. However, the rate of prevalence varies based on the definition and study context, ranging from 5% to 30% ( Beers *et al.*, 2003). A recent report showed that 18.8% of children in the United States had special health care needs ( Ghandour et al., 2022).

*2. Discussion. I would report in more detail which programs or interventions suggested for helping teachers in facing the burdens due to interacting with CCC children.*

**Response:**

 Unfortunately, we could not find any specific program for teachers of children with CCC. Based on Comments 2 and 4, we have added a discussion of the potential contributions of motivating their practice.

 In Discussion section,

 Unfortunately, a validated program for improving interaction with CCC for special education teachers is not widely available. Self-determination theory, a theory of human motivation that has been applied across research fields, posits three basic psychological needs as being necessary for psychological health and well-being: autonomy, competence, and relatedness ( Deci and Ryan, 1980; Deci et al., 2017; Ryan & Deci, 2020). Based on this framework, one study reported that teachers experiencing higher relatedness with students engaged more in work and show less emotional exhaustion ( Klassen et al., 2012). Although there are limitations to the application of self-determination theory in special education, it may be useful to explore the satisfaction or frustration of teachers’ basic psychological needs based on this theoretical framework devising support measures.

*3. Generalization: They found those results in a specific context. Do the AA think they can be applied worldwide?*

**Response:**

 The findings are based on interviews with Japanese teachers within a certain educational context; therefore, the generalizability of the findings is more context-dependent, rather than being generalizable. However, future comparisons with other cultures or contexts may provide meaningful insights into the current findings.

 We have clarified the context in the summary of the results (Discussion section) and Conclusions, and discussed it in the Limitations section.

 In Discussion section,

 Focusing on areas of interaction and communication, this study investigated current perceptions, experiences, and educational challenges encountered by special education teachers who work with children with CCC in Japan.

 In Limitations section,

 Additionally, future comparisons between other countries or meta-synthesis of studies could yield meaningful insights into the current findings.

 In Conclusion section,

 This study conducted interviews with Japanese special education teachers to investigate the qualitative aspects of experiences with children with severe CCC.

*4. Overall. I would refer to a specific theory. Maybe the SDT theory regarding the autonomy-support vs. control could help (e.g., Ryan & Deci, 2020) because it states different supportive or less functional modalities (e.g., Moè et al., 2022). Referring to self-efficacy could be a further option (e.g., Kasalak & Dagyar, 2020).*

*Suggested references:*

*Kasalak, G., & Dagyar, M. (2020). The relationship between teacher self-efficacy and teacher job satisfaction: A meta-analysis of the teaching and learning international survey (TALIS). Educational Sciences: Theory and Practice, 20(3), 16-33.*

*Moè, A., Consiglio, P., & Katz, I. (2022). Exploring the circumplex model of motivating and demotivating teaching styles: The role of teacher need satisfaction and need frustration. Teaching and Teacher Education, 118, 103823.*

*Ryan, R. M., & Deci, E. L. (2020). Intrinsic and extrinsic motivation from a self determination theory perspective: Definitions, theory, practices, and future directions. Contemporary Educational Psychology, 101860.*

*I wish the AA the best with their research!*

**Response:**

 Thank you for the suggestion. Based on the reviewer’s comment, we have discussed the findings with reference to self-determination theory.

 In Discussion section,

 Unfortunately, a validated program for improving interaction with CCC for special education teachers is not widely available. Self-determination theory, a theory of human motivation that has been applied across research fields, posits three basic psychological needs as being necessary for psychological health and well-being: autonomy, competence, and relatedness ( Deci and Ryan, 1980; Deci et al., 2017; Ryan & Deci, 2020). Based on this framework, one study reported that teachers experiencing higher relatedness with students engaged more in work and show less emotional exhaustion ( Klassen et al., 2012). Although there are limitations to the application of self-determination theory in special education, it may be useful to explore the satisfaction or frustration of teachers’ basic psychological needs based on this theoretical framework devising support measures.

 In Implication for practice section,

 As discussed above, satisfaction or frustration of basic psychological needs (i.e., autonomy, competence, and relatedness) may be essential factors in understanding the experience of teachers and for supervisors to provide support ( Moè et al., 2022; Ryan & Deci, 2020).

# Reviewer report: Reviewer response for version 1

**DOI:** 10.5256/f1000research.141784.r209533

**Published:** 2023-10-09

**Authors:** Lina Diaz-Castro

**Affiliations:** 1 National Institute of Psychiatry Ramón de la Fuente Muñiz, Mexico City, Mexico

Dear Author,

 I am sending you the review of the article "Experiences and Challenges of Special Education Teachers in Supporting Children with Complex Chronic Conditions: A Qualitative Study".

***General comments:***

 The study investigates the experiences and challenges faced by special education teachers in Japan when providing support to children with complex chronic conditions (CCC). It employs a qualitative research design involving semi-structured face-to-face interviews with 21 special education teachers who work with sick children. The study identifies four main themes: "searching for the meaning," "CCC as a difficult reality," "widening experience for the future," and "priority for interacting with children." These themes reveal the complexities of teacher-child interactions, the unique challenges posed by CCC, the importance of widening children's experiences, and the significance of building positive relationships with these children.

 Replicability and Generalizability:

 Replicability: The study provides a detailed description of its methods, including data collection and analysis, which makes it replicable for researchers interested in conducting similar qualitative studies.

 Generalizability: The generalizability of the study's findings may be limited by the specific context of Japanese special education schools for children with CCC. Further research in diverse settings and with larger sample sizes would enhance the generalizability of the findings.

 In summary, the article presents valuable insights into the experiences and challenges faced by special education teachers when working with children with CCC. To enhance the scientific soundness of the article, the authors should consider the following:

  

- Improve the clarity of certain sections, particularly those related to the thematic analysis process.
- Clarify details of the data analysis, including the coding process.
- Consider making the source data underlying the results available for reproducibility.
- Encourage further research in diverse settings to enhance the generalizability of findings.
- The study contributes to the understanding of teacher-child interactions in the context of special education for children with complex chronic conditions and offers important implications for teacher training and support in this field.

**Specific comments**

**Is the work clearly and accurately presented and does it cite the current literature?**

 This work demonstrates clarity, accuracy, and appropriate citation of relevant literature up to my knowledge cutoff date. The article reads well and maintains a logical flow. However, consider using subheadings within sections to break up the text further and improve the readability.

**Is the study design appropriate and is the work technically sound?**

 The study's design and methodology appear to be well-suited to its research objectives and maintain technical soundness, based on the provided information.

**Are sufficient details of methods and analysis provided to allow replication by others?**

 In my opinion, additional information is typically required in a research study to facilitate replication:

 Sampling Details: While the study mentions inclusion criteria for participants, it's helpful to provide more information about how participants were selected, and recruited, and any potential biases in the selection process.

 Interview Guide: The study mentions the use of an interview guide but does not provide the full list of questions or prompts included in the guide. Sharing the complete guide would help others understand how the interviews were conducted and structured.

 Data Collection Procedures: Detailed information on the interview process, including how interviews were scheduled, conducted, and audio-recorded, is essential for replication. Additionally, information on any piloting or pre-testing of the interview guide should be included.

 Data Analysis: While the study mentions thematic analysis and the use of MAXQDA software, it lacks a step-by-step description of the analytical process. Replicators would typically need to know how codes were generated, how themes were identified, and the criteria used for theme selection.

**If applicable, is the statistical analysis and its interpretation appropriate?**

 The study does not mention the use of statistical analysis.

 The description of the thematic analysis process is well-detailed, and it's good that the authors followed an inductive approach without preconceived codes. Mentioning the software (MAXQDA) used for data analysis is helpful. Authors might want to provide a brief explanation of the number of coders involved and their qualifications to ensure the rigor of the analysis.

 Translation: The authors mentioned that excerpts were translated into English for presentation in the article. It's important to acknowledge that translation can introduce some degree of interpretation bias. Mentioning that a qualified translator, preferably bilingual, was involved in the process can help address potential concerns about translation accuracy.

 Authors might want to provide a bit more detail about the roles each author played in the research process, especially in data analysis.

**Are all the source data underlying the results available to ensure full reproducibility?**

 The provided text does not indicate whether the source data underlying the results of this study are available for full reproducibility. In many cases, qualitative research studies may not make their raw data, such as interview transcripts, publicly available to protect the privacy and confidentiality of study participants.

 However, it is common practice in qualitative research to provide detailed descriptions of the research methods, including the process of data collection (interviews in this case), coding procedures, and thematic analysis, so that other researchers can understand how the results were derived and potentially replicate the study using their own data if desired.

**Are the conclusions drawn adequately supported by the results?**

 The conclusions drawn in the study appear to be adequately supported by the results and findings presented. The study conducted interviews with special education teachers and identified key themes related to their experiences and challenges when working with children with complex chronic conditions (CCC).

Is the work clearly and accurately presented and does it cite the current literature?

Yes

If applicable, is the statistical analysis and its interpretation appropriate?

Not applicable

Are all the source data underlying the results available to ensure full reproducibility?

Partly

Is the study design appropriate and is the work technically sound?

Yes

Are the conclusions drawn adequately supported by the results?

Yes

Are sufficient details of methods and analysis provided to allow replication by others?

Partly

Reviewer Expertise:

Mental Disorderds. /  Public Health. / Research in Health Systems.

I confirm that I have read this submission and believe that I have an appropriate level of expertise to confirm that it is of an acceptable scientific standard.

# Response

**Published:** 2023-12-12

**Authors:** Haruo Fujino

**Affiliations:** Osaka University, Japan

**Reviewer Report**

*09 Oct 2023 | for Version 1*

*Lina Diaz-Castro, National Institute of Psychiatry Ramón de la Fuente Muñiz, Mexico City, Mexico*

*Dear Author,*

*I am sending you the review of the article "Experiences and Challenges of Special Education Teachers in Supporting Children with Complex Chronic Conditions: A Qualitative Study".*

*General comments:*

*The study investigates the experiences and challenges faced by special education teachers in Japan when providing support to children with complex chronic conditions (CCC). It employs a qualitative research design involving semi-structured face-to-face interviews with 21 special education teachers who work with sick children. The study identifies four main themes: "searching for the meaning," "CCC as a difficult reality," "widening experience for the future," and "priority for interacting with children." These themes reveal the complexities of teacher-child interactions, the unique challenges posed by CCC, the importance of widening children's experiences, and the significance of building positive relationships with these children.*

*Replicability and Generalizability:*

*Replicability: The study provides a detailed description of its methods, including data collection and analysis, which makes it replicable for researchers interested in conducting similar qualitative studies.*

*Generalizability: The generalizability of the study's findings may be limited by the specific context of Japanese special education schools for children with CCC. Further research in diverse settings and with larger sample sizes would enhance the generalizability of the findings.*

*In summary, the article presents valuable insights into the experiences and challenges faced by special education teachers when working with children with CCC. To enhance the scientific soundness of the article, the authors should consider the following:*

*Improve the clarity of certain sections, particularly those related to the thematic analysis process.*

*Clarify details of the data analysis, including the coding process.*

*Consider making the source data underlying the results available for reproducibility.*

*Encourage further research in diverse settings to enhance the generalizability of findings.*

*The study contributes to the understanding of teacher-child interactions in the context of special education for children with complex chronic conditions and offers important implications for teacher training and support in this field.*

**Response:**

 Thank you for considering our manuscript. Based on the reviewers’ comments, we have revised the manuscript. Please refer to our responses for the specific comments.

*Specific comments*

*Is the work clearly and accurately presented and does it cite the current literature?*

*1. This work demonstrates clarity, accuracy, and appropriate citation of relevant literature up to my knowledge cutoff date. The article reads well and maintains a logical flow. However, consider using subheadings within sections to break up the text further and improve the readability.*

***Response:***

 Thank you for your suggestions. We have added subheadings in the manuscript to improve readability.

*Is the study design appropriate and is the work technically sound?*

*2. The study's design and methodology appear to be well-suited to its research objectives and maintain technical soundness, based on the provided information.*

**Response:**

 Thank you for your positive evaluation of our study.

*Are sufficient details of methods and analysis provided to allow replication by others?*

*3. In my opinion, additional information is typically required in a research study to facilitate replication:*

*3-1. Sampling Details: While the study mentions inclusion criteria for participants, it's helpful to provide more information about how participants were selected, and recruited, and any potential biases in the selection process.*

**Response:**

 Based on the reviewer’s suggestion, we have included additional details in the Participants section and discussed potential biases in the Limitations section.

 In Participants section,

 The potential participant teachers were selected by the school principals or a senior teacher based on the teachers’ experience and backgrounds. Teachers with minimal experience in this area were excluded.

 In Limitations section,

 This issue may be also related to the method by which the teachers were recruited. Since the participants were selected by school principals or a senior teacher, the sample of this study might be biased to teachers who were likely to be more capable. Inclusion of participants with wider experience and capabilities may expand the rage of their experiences.

*3-2. Interview Guide: The study mentions the use of an interview guide but does not provide the full list of questions or prompts included in the guide. Sharing the complete guide would help others understand how the interviews were conducted and structured.*

**Response**

 We have shared this information in the Extended Data section (OSF repository, https://www.doi.org/10.17605/OSF.IO/WQNFV).

*3-3. Data Collection Procedures: Detailed information on the interview process, including how interviews were scheduled, conducted, and audio-recorded, is essential for replication. Additionally, information on any piloting or pre-testing of the interview guide should be included.*

**Response**

 We have included further details of these procedures. The interview guide was not pilot tested before the interview; it was developed by two of the authors, who have different professional backgrounds.

 In Procedures section,

 The interview-guide was developed by two researchers (first and third authors), who had different professions. However, it was not performed in this study. Although we used this guide to elicit experiential narratives, we also encouraged the participants to talk freely about any relevant views, ideas, experiences, and challenges.

*3-3. Data Analysis: While the study mentions thematic analysis and the use of MAXQDA software, it lacks a step-by-step description of the analytical process. Replicators would typically need to know how codes were generated, how themes were identified, and the criteria used for theme selection.*

**Response:**

 Based on the reviewer’s comment, we included this information in the analysis section.

 In Data analysis section,

 Before the data were coded, impressions of the interview transcripts were discussed between the first and third authors. In the coding process, the authors tried to create codes that summarized the data meaning based on the research questions of this study. The first author coded the transcribed text, then discussed the coding with the second author. Because the codes were modified during this discussion, the first author re-coded the original text to confirm the data context. Only the first author coded all the transcripts based on the approach employed in thematic analysis ( Braun & Clarke, 2021; Clarke & Braun, 2013). The themes were developed by repeatedly reviewing the content of the code, creating tentative themes, comparing the theme to the code, and repeating the review to ensure that each theme reflected a central organizing concept ( Clarke & Braun, 2013).

*If applicable, is the statistical analysis and its interpretation appropriate?*

*4. The study does not mention the use of statistical analysis.*

*The description of the thematic analysis process is well-detailed, and it's good that the authors followed an inductive approach without preconceived codes. Mentioning the software (MAXQDA) used for data analysis is helpful. Authors might want to provide a brief explanation of the number of coders involved and their qualifications to ensure the rigor of the analysis.*

**Response:**

 Thank you for your suggestions. Accordingly, we have added a short explanation of the coder and the approach of the thematic analysis in the Analysis section.

 In Data analysis section,

 Only the first author coded all the transcripts based on the approach employed in thematic analysis ( Braun & Clarke, 2021; Clarke & Braun, 2013).

*5. Translation: The authors mentioned that excerpts were translated into English for presentation in the article. It's important to acknowledge that translation can introduce some degree of interpretation bias. Mentioning that a qualified translator, preferably bilingual, was involved in the process can help address potential concerns about translation accuracy.*

**Response:**

 We have added information about the translator in the manuscript.

 In Data analysis section,

 After completing the qualitative data analysis in Japanese (the original language of the data), the excerpts were translated into English for presentation in this article by a professional bilingual translator.

*6. Authors might want to provide a bit more detail about the roles each author played in the research process, especially in data analysis.*

**Response:**

 We have clarified the roles of the authors in the data analysis section.

 In Data analysis section,

 Before the data were coded, impressions of the interview transcripts were discussed between the first and third authors. In the coding process, the authors tried to create codes that summarized the data meaning based on the research questions of this study. The first author coded the transcribed text, then discussed the coding with the second author. Because the codes were modified during this discussion, the first author re-coded the original text to confirm the data context. Only the first author coded all the transcripts based on the approach employed in thematic analysis ( Braun & Clarke, 2021; Clarke & Braun, 2013). The themes were developed by repeatedly reviewing the content of the code, creating tentative themes, comparing the theme to the code, and repeating the review to ensure that each theme reflected a central organizing concept ( Clarke & Braun, 2013).

*Are all the source data underlying the results available to ensure full reproducibility?*

*7. The provided text does not indicate whether the source data underlying the results of this study are available for full reproducibility. In many cases, qualitative research studies may not make their raw data, such as interview transcripts, publicly available to protect the privacy and confidentiality of study participants.*

*However, it is common practice in qualitative research to provide detailed descriptions of the research methods, including the process of data collection (interviews in this case), coding procedures, and thematic analysis, so that other researchers can understand how the results were derived and potentially replicate the study using their own data if desired.*

**Response:**

 Thank you for your understanding. We have revised the section and added more detailed information in the Methods section. Please also refer to our responses to Comments 3-3, 4, and 6.

*Are the conclusions drawn adequately supported by the results?*

*8. The conclusions drawn in the study appear to be adequately supported by the results and findings presented. The study conducted interviews with special education teachers and identified key themes related to their experiences and challenges when working with children with complex chronic conditions (CCC).*

**Response:**

 Thank you for your comment.
